# Effect of Biliary Drainage on the Toxicity and Toxicokinetics of *Amanita exitialis* in Beagles

**DOI:** 10.3390/toxins10060215

**Published:** 2018-05-25

**Authors:** Jian Sun, Yu-Tao Zhang, Yu-Min Niu, Hai-Jiao Li, Yu Yin, Yi-Zhe Zhang, Pei-Bin Ma, Jing Zhou, Jun-Jia Lu, Hong-Shun Zhang, Cheng-Ye Sun

**Affiliations:** 1 National Institute of Occupational Health and Poison Control, Chinese Centre for Disease Control and Prevention, Beijing 100050, China; sunjian2019652@163.com (J.S.); zhangyutao0708@163.com (Y.-T.Z.); lihaijiao715@163.com (H.-J.L.); yymssyn@126.com (Y.Y.); zyz97@263.net (Y.-Z.Z.); mapb@niohp.chinacdc.cn (P.-B.M.); zhoujing@niohp.chinacdc.cn (J.Z.); lujunjia1990@163.com (J.-J.L.); 2 Institute of Public Health and Management, Ningxia Medical University, Yinchuan 750004, China; 3 Beijing Key Laboratory of Diagnostic and Traceability Technologies for Food Poisoning, Beijing Center for Disease Control and Prevention, Beijing 100013, China; nym0542010@163.com

**Keywords:** *Amanita exitialis*, amatoxins, beagles, bile drainage, urine

## Abstract

Amatoxin poisoning induces delayed-onset acute liver failure, which are responsible for more than 90% of deaths in mushroom poisoning. It has been postulated from animal and human studies that biliary drainage interrupting enterohepatic amatoxin circulation may affect amatoxin poisoning. Dogs were randomly divided into four groups of six animals each. In 20 mg/kg and 60 mg/kg with biliary drainage groups, after accepting bile drainage operation, beagles were fed *Amanita exitialis* powder (20 or 60 mg/kg) in starch capsules. In control and bile drainage groups, the beagle dogs were fed with empty capsules. They were assessed for toxicity signs, biochemical and pathological changes, and peptide toxins in plasma, urine and bile. The data were directly compared with those from our published studies on *Amanita exitialis*-exposed beagles without biliary drainage. Amatoxins were rapidly absorbed and eliminated from plasma after *Amanita exitialis* ingestion. Amatoxins in 0–1-day urine accounted for more than 90% of the total urine excretion, and amatoxins in bile accounted for less than 20% of the total urine and bile excretion. The dogs with biliary drainage showed less severe toxicity signs and biochemical and pathological changes and much lower internal exposure than dogs without biliary drainage. Biliary drainage caused a more than 70% reduction in intestinal amatoxin absorption and could reduce amatoxin absorption from the gastrointestinal tract.

## 1. Introduction

According to the Chinese Management Information System of Public Health Emergency, 3701 patients and 786 deaths were reported in 576 incidents of mushroom poisoning from 2004 to 2014 [1]. Mushroom poisoning is considered the main cause of food poisoning-related mortality in China. The section of *Phalloideae* in *Amanita* genus has been responsible for at least 70% of deaths from mushroom poisoning incidents in China [2], and the hepatotoxic effects of amatoxins and phallotoxins from *Amanita* species [3] are well known [4]. Amatoxins bind to the Retinol Binding Protein 1 (RBP1) of RNA polymerase II to form a complex, and thereby progressively decrease the mRNA content, which causes inhibition of protein synthesis and eventually results in cell death [5]. Phallotoxins and virotoxins firmly bind to F-actin. It has been postulated that cross-linking effect by bifunctionality of the phallotoxin molecules or allostery of enforcing the coherence between actin units must play an important role in their stabilizing action [6]. Lesions are found particularly in the liver [7].

At 6–24 h after ingestion of amatoxin-containing mushroom, patients present gastrointestinal symptoms including nausea, vomiting, abdominal pain, and diarrhea [8,9]. At 36–48 h after ingestion, biochemical signs of liver damage in the patients increase, especially in liver transaminase and bilirubin levels. Three to four days after ingestion, patients may develop jaundice, slight enlargement and tenderness of the liver. Acute hepatic failure, such as hyperbilirubinemia, coagulation disorders, bleeding, and encephalopathy, may develop in the following days. Death may occur within 3–7 days after mushroom ingestion, while surviving patients recover within 14–21 days [3,10,11]. Based on animal studies, it has been postulated that intravenously administered amanitin is excreted in the bile fluid [12,13]. Biliary amanitin excretion prolongs its presence in enterohepatic and systemic circulation because of intestinal reuptake. This mechanism could significantly influence the clinical course of amatoxins poisoning. Based on this theory, interruption of enterohepatic circulation has become an alternative part of detoxification methods. Recently, endoscopic nasobiliary drainage was performed in a patient who ingested *Amanita bisporigera*. A total of 2.5 mg of amatoxin was removed through bile fluid on Day 2 after ingestion; however, the relevance between bile drainage and clinical outcome remained unclear [14]. Further, amatoxin kinetics in human poisoning cases remain to be elucidated because of delayed clinical presentations of most intoxicated patients.

In previous studies, the radioactivity of labeled amatoxins in the serum of dogs or pigs was determined after an intravenous injection of amatoxins; bile was collected and measured using tubes tied to the bile duct under general anesthesia [13,15]. More than 80% of 14C-methyl-γ-amanitin (14C-A) was eliminated in the urine, with less than 10% in the bile [15]. Different toxin exposure patterns might result in different toxicokinetic data. 14C-A and 3H-O-methyl-dehydroxymethyl-α-amanitin (3H-A) were used for toxicokinetic studies in the animals; the animals were under general anesthesia during the experiments. The bile and urine excretion data obtained were different from those of animals that orally ingested α-amanitin and γ-amanitin.

In our previous study, the toxicity of *Amanita exitialis* and the toxicokinetics of its constituent peptide toxins were determined in dogs that were orally administered 20 and 60 mg/kg *Amanita exitialis* [16,17]. This model induced by *Amanita exitialis* is consistent with clinical pathophysiological process of acute toxic liver failure induced by mushrooms containing peptide toxins [17]. In the present study, we established a long-term bile drainage model to evaluate biliary and urinary amatoxin excretion in 20 and 60 mg/kg *Amanita exitialis*-exposed beagles. Furthermore, we studied the effect of biliary drainage on the toxicity and toxicokinetics of *Amanita exitialis* by directly comparing the data obtained with those from our published studies on beagles with the exposure of the same dose of *Amanita exitialis* without bile drainage.

## 2. Results

### 2.1. Peptide Toxins in Amanita exitialis

The total and average concentrations of the four peptide toxins in the mushroom are shown in Table 1. The amount of peptide toxins from *Amanita exitialis* ingested in different experimental groups is presented in Table S1.

### 2.2. Signs of Toxicity

The dogs were clinically back to normal on Day 6 after accepting biliary drainage operation. After exposure, no changes were noted in the control, biliary drainage, and 20 mg/kg with biliary drainage groups. However, vomiting, diarrhea, and a loss of appetite were observed at 12–48 h in the 60 mg/kg with biliary drainage group. No animal died during the experiment (Table 2).

### 2.3. Blood Biochemistry Analysis

Alanine transaminase (ALT), aspartate aminotransferase (AST), and alkaline phosphatase (ALP) levels slightly increased and then normalized at Day 7 after the surgery. Total bilirubin (TBIL), direct bilirubin (DBIL), prothrombin time (PT), activated partial thromboplastin time (APTT), the international normalized ratio (INR), creatinine, and blood urea nitrogen (BUN) were normal after the surgery (Table S2).

**Figure 1:**
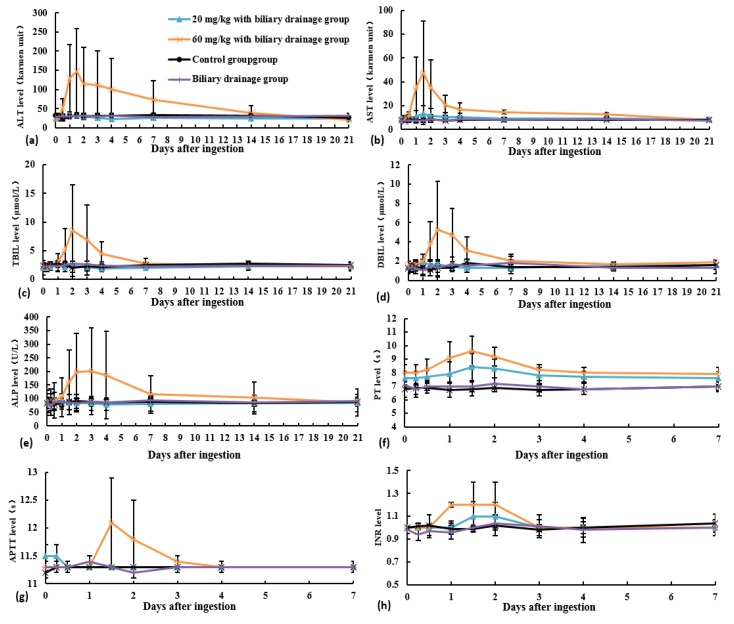
Blood chemistry profiles of beagles in different experimental groups: (**a**) ALT level; (**b**) AST level; (**c**) TBIL level; (**d**) DBIL level; (**e**) ALP level; (**f**) PT level; (**g**) APTT level; and (**h**) INR level.

**Figure 2:**
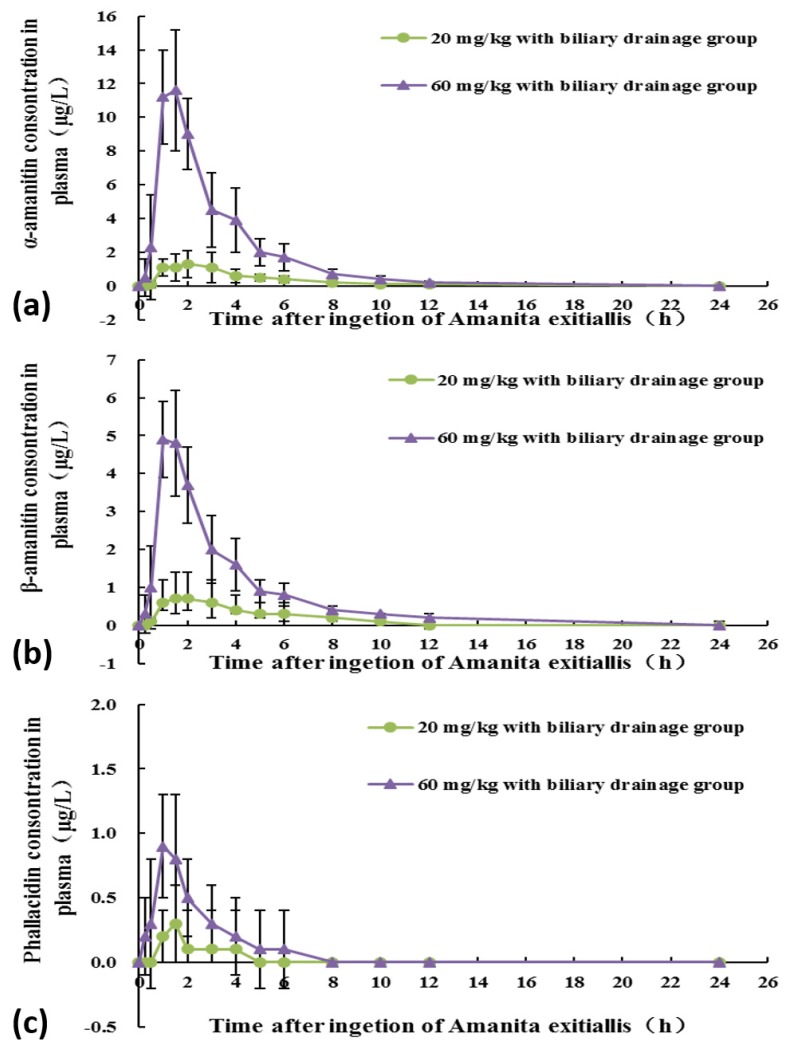
Dynamic changes of peptide toxins in the blood of beagles in different experimental group: (**a**) α-amanitin levels; (**b**) β-amanitin levels; and (**c**) phallacidin levels.

In the control and biliary drainage groups, ALT, AST, TBIL, DBIL, ALP, PT, APTT, INR, creatinine, and BUN were in the normal range (Figure 1 and Figure 2). In the 20 mg/kg with biliary drainage group, ALT and AST levels, PT, and INR slightly increased and then normalized at Day 7 after *Amanita exitialis* ingestion. The peak ALT, AST and PT levels were 34.5 U/L, 13.4 U/L, and 8.4 s, respectively. During the experiment, TBIL, DBIL, ALP, and APTT were in the normal range (Figure 1 and Figure 2).

In the 60 mg/kg with biliary drainage group, ALT, AST, TBIL, DBIL, and ALP levels along with PT, APTT, and INR began to increase at Day 1 after *Amanita exitialis* ingestion. Peak ALT, AST, PT, APTT, and INR values were observed on Day 1.5 and were 147.3 U/L, 47.6 U/L, 9.6 s, 12.1 s, and 1.2, respectively. Peak TBIL and DBIL levels (8.5 μmol/L and 5.3 μmol/L, respectively) were observed on Day 2. The peak ALP levels (201.2 U/L) was observed at Day 3 after *Amanita exitialis* ingestion. After reaching their peaks, these values began to decline and normalized at Day 21 after *Amanita exitialis* ingestion (Figure 1). Creatinine and BUN values were normal during the experiment in both dose groups.

### 2.4. Histopathological Examination

With an overdose of sodium pentobarbital, one beagle from each group was euthanized on the third day after ingestion of *Amanita exitialis*. Necropsy was performed and liver tissues were taken for histopathological examination. A general pathological examination of the liver showed that the color was reddish-brown and the surface was smooth (Figure S1). The hepatic tissue structure was normal, and the hepatic lobules structure was clear. A microscopic examination of the hepatic tissue revealed no obvious hepatocellular necrosis, red blood cells, inflammatory cell infiltration, or fibrous hyperplasia (Figures S2 and S3). No obvious lesions were found in the kidney, heart, lung, stomach, urinary bladder, spleen, small intestine, colon, or pancreas.

### 2.5. Peptide Toxins in the Blood

The dynamic changes of the peptide toxins in the 20 and 60 mg/kg with biliary drainage groups are shown in Figure 2. The detection rate of peptide toxins in the blood of the beagles is shown in Table S3. The calculated toxicokinetic parameters are shown in Table 3.

AUC, and C_max_ values in the 60 mg/kg with biliary drainage group were statistically higher than these values in the 20 mg/kg with biliary drainage group. Furthermore, there were no significant differences in T_1/2_, T_max_, clearance (CL/F), V_z_/F, and mean residence time (MRT) values between the two groups. In the 20 mg/kg with biliary drainage group, there was no statistically significant difference in any of the toxicokinetic parameters between α-amanitin and β-amanitin. However, in the 60 mg/kg with biliary drainage group, AUC and C_max_ values for α-amanitin were statistically higher than these values for β-amanitin. Furthermore, except for the T_max_ value, other toxicokinetic parameters for phallacidin were significantly (*p* ≤ 0.05) different from the respective values for α- and β-amanitin.

### 2.6. Peptide Toxins in the Urine

The urine volume of the experimental groups is presented in Figure S4a. In the 20 mg/kg with biliary drainage group, α-amanitin, β-amanitin, and phallacidin were detected within two days. In the 60 mg/kg with biliary drainage group, α-amanitin and phallacidin were detected within three days, and β-amanitin was detected within two days (Table S4). The detection rate of peptide toxins in the urine is presented in Table S5. In the 20 mg/kg with biliary drainage group, the accumulated amounts of α-amanitin, β-amanitin, and phallacidin were 0.0151, 0.0055, and 0.0014 mg, respectively. In the 60 mg/kg with biliary drainage group, the corresponding accumulated amounts were 0.0222, 0.0068, and 0.0025 mg. In both the 20 and 60 mg/kg with biliary drainage groups, more than 90% of the total urine excretion of α-amanitin and β-amanitin occurred on Days 0–1. More than 90% of the total urine excretion of phallacidin occurred on Days 0–1 in the 20 mg/kg with biliary drainage group, while, for the 60 mg/kg with biliary drainage group, 70.5% of the urine excretion of phallotoxin occurred on Days 0–1 and 22.1% on Days 1–2 (Table 4).

### 2.7. Peptide Toxins in the Bile

The bile volumes of the experimental groups are presented in Figure S4b. In the 20 mg/kg with biliary drainage group, α-amanitin and β-amanitin were detected within one day, whereas phallacidin was not detected. In the 60 mg/kg with biliary drainage group, α-amanitin, β-amanitin, and phallacidin were detected within one day (Table S6). The detection rate of peptide toxins in the bile of beagles is presented in Table S7. In the 20 mg/kg with biliary drainage group, the accumulated amounts of α-amanitin, β-amanitin, and phallacidin were 0.00007, 0.00014, and 0.00005 mg, respectively. In the 60 mg/kg with biliary drainage group, the corresponding accumulated amounts were 0.00061, 0.0012, and 0.0002 mg. In both the 20 and 60 mg/kg with biliary drainage groups, 100% of the total bile excretion of alpha-and beta amanitin occurred on Days 0–1. One hundred percent of the total bile excretion of phallacidin occurred on Days 0–1 in the 20 mg/kg with biliary drainage group, while for the 60 mg/kg with biliary drainage group, 81.7% of the bile excretion of phallotoxin occurred on Days 0–1 and 18.3% on Days 1–2 (Table 5).

In the 20 mg/kg with biliary drainage group, α-amanitin, β-amanitin, and phallacidin in the bile accounted for less than 10% of the total excretion in urine and bile. In the 60 mg/kg with biliary drainage group, α-amanitin, β-amanitin, and phallacidin in the bile accounted for less than 20% of the total excretion in urine and bile (Table 6).

## 3. Discussion

In our previous study, the average concentrations of the different toxins in *Amanita exitialis* powder were as follows: α-amanitin (1945.4 ± 91.1) mg/kg > β-amanitin (921.6 ± 58.8) mg/kg > phallacidin (607.2 ± 40) mg/kg > γ-amanitin (8.3 ± 0.2) mg/kg [17]. In the present study, the average concentrations of the different toxins in the same *Amanita exitialis* powder were as follows: α-amanitin (1966.1 ± 64.6) mg/kg > β-amanitin (915.1 ± 76.9) mg/kg > phallacidin (602.3 ± 63.1) mg/kg > γ-amanitin (8.5 ± 0.2) mg/kg. The quantity of peptide toxins in *Amanita exitialis* powder did not change in a year. Further, amatoxins cannot be removed or destroyed by cooking due to its thermostability [3]. In September 2016, a Sina website reported that seven people in Ning Bo Province were poisoned with dried wild mushroom, which was from Yun Nan Province of China [18]. This suggests that dried wild mushroom ingestion also poses a risk of poisoning with amatoxins.

In our previous study, 20 or 60 mg/kg *Amanita exitialis* powder was fed to beagles in starch capsules, and then the beagles were assessed for toxicity signs and biochemical and pathological changes. Vomiting and diarrhea were observed in the beagles at 12–48 h following *Amanita exitialis* ingestion. Furthermore, plasma ALT and AST levels, PT, and APTT peaked at 36 h after *Amanita exitialis* ingestion. TBIL, DBIL, and ALP levels peaked at 48 h after *Amanita exitialis* ingestion. Three dogs died between 24 h and 72 h after ingestion of 60 mg/kg *Amanita exitialis*. Additionally, histopathological examinations of the liver showed hemorrhagic hepatocyte necrosis [16,17]. In the present study, 20 or 60 mg/kg *Amanita exitialis* powder was fed to beagles, on the eighth day after biliary drainage operation. These two studies showed us that, after *Amanita exitialis* ingestion at the same dose, the dogs with biliary drainage showed less severe toxicity signs than did those without biliary drainage. Dynamic changes in ALT, AST, TBIL, DBIL, and ALP levels, PT, APTT, and INR in beagles in the different experimental groups in the two studies are presented in Figures S5 and S6. The findings suggest that biliary drainage might play a role in *Amanita exitialis* poisoning.

A plasma kinetics study of amatoxins in humans showed that amatoxins disappeared rapidly from the plasma. Vesconi et al. found amatoxins were detectable in the serum of 65% patients within 30 h after ingestion of *Amanita phalloides* [19]. In another study in patients intoxicated with *Amanita phalloides*, amatoxins were detectable in the plasma of most patients within 36 h after ingestion [20]. Our study showed that α-amanitin, β-amanitin, and phallacidin are rapidly absorbed into systemic circulation after *Amanita exitialis* ingestion. These toxins were rapidly detected (30 min) in plasma and eliminated after *Amanita exitialis* ingestion. The T_1/2_ of α-amanitin and β-amanitin in the 20 mg/kg with biliary drainage group was 1.08 ± 0.76 and 1.29 ± 1.07 h, respectively. The corresponding values for the 60 mg/kg with biliary drainage group were 1.2 ± 0.58 and 1.54 ± 0.71 h, whereas T_1/2_ of phallacidin was 0.62 ± 0.32 h. The values of AUC, and C_max_ in the 60 mg/kg with biliary drainage group were higher than the respective values in the 20 mg/kg with biliary drainage group. This indicated that an increase in the external exposure concentration (amatoxins in *Amanita exitialis*) resulted in a corresponding increase in the internal exposure concentration (amatoxins in plasma). In the 60 mg/kg with biliary drainage group, the CL/F, V_z_/F, and MRT values for α-amanitin and β-amanitin were significantly different from the respective values for phallacidin. This may be explained by the chemical structures differences between amatoxins (bicyclic octapeptides) and phallotoxins (bicyclic heptapeptides). Unlike in our previous study, in this study, after ingestion of *Amanita exitialis* at the same dose, the dogs with biliary drainage showed much lower AUC, and C_max_ values than dogs without biliary drainage [16]. Dynamic changes in plasma amatoxin concentrations among beagles in different experimental groups in the two studies are shown in Figure S7. At the same exposure dose, the dogs with biliary drainage showed much lower internal exposure (area under the curve of amatoxins in plasma) than dogs without biliary drainage [16]. This indicates that bile plays a promoting effect on the intestinal absorption of amatoxins. Biliary drainage caused a more than 70% reduction in intestinal amatoxin absorption. Similar findings were observed for water-soluble vitamin B_12_ in human and rats. Teo et al. found that bile exclusion caused a 50–60% reduction in vitamin B_12_ absorption from the intestinal lumen [21]. However, the mechanism by which bile enhances amatoxin absorption is unknown. Bile contains a small amount of bicarbonate compared to those in pancreatic juice, and the quantity of calcium in bile is not greater than that in gastric or pancreatic juice [22]. Therefore, intestinal pH and calcium ion concentration are probably not relevant to the effect of bile as described [23]. The mechanism by which bile enhances amatoxin absorption may be explained as follows: amatoxins normally bind to intrinsic factors in the stomach. It is reported that the presence of excess free intrinsic factors inhibits the combination of the intrinsic factor-bound amatoxins to the ileal mucosa. The combination of bile acids to free intrinsic factors in the upper intestine might prevent the inhibition that would otherwise occur. Alternatively, bile acids may cause dissociation of the amatoxin-intrinsic factor complex at the given receptor site, thus enhancing amatoxin absorption [24,25,26]. However, there is little evidence to support the above hypothesis.

Previous studies on amatoxins in the urine of patients and beagles showed that amatoxins were detectable in the urine within four days after ingestion [20,27]. Our present results showed that amatoxin levels in the urine of beagles with biliary drainage were the highest on Day 1 and persisted in some beagles for up to three days after *Amanita exitialis* ingestion. The difference in detectable time in the urine can be explained by the low level of internal exposure (amatoxins in the blood). Our results in beagles with biliary drainage also showed that α-amanitin, β-amanitin, and phallacidin in the 0–2-day urine accounted for more than 90% (more than 95% for α-amanitin and β-amanitin) of the total urine excretion. These findings agree with those of our previous studies in dogs without biliary drainage, indicating that biliary drainage had no effect on urine excretion of amatoxins in beagles. Our previous study showed that in the 20 mg/kg with no biliary drainage group, the accumulated amounts of α-amanitin, β-amanitin, and phallacidin were 0.039, 0.0157, and 0.0037 mg, respectively (Table S6). In the 60 mg/kg with biliary drainage group, the corresponding accumulated amounts were 0.063, 0.0264, and 0.0118 mg (Table S8). At the same exposure dose, the accumulated amounts of peptide toxins in the urine of dogs with biliary drainage were lower than those in the dogs without biliary drainage.

Our present study showed that, in beagles in the biliary drainage groups, α-amanitin and β-amanitin were detected within one day and phallacidin was detected within two days. Busi et al. found low amatoxin concentrations in samples of gastroduodenal fluid obtained by nasogastric aspiration in five patients within 48 h after ingestion of *Amanita phalloides* [28]. Jaeger et al. studied 12 patients with *Amanita phalloides* poisoning and found α-amanitin and β-amanitin in the gastroduodenal fluid of three patients at 34–84 h after ingestion [20]. The difference in detectable time in bile between beagles and patients may be explained by the distinct levels of internal exposure (amatoxins in the blood) between animals and patients. To study amatoxins in bile, bile was collected from animals intravenously injected with labeled amatoxins through tubes tied to the bile duct under general anesthesia and measured. The recovery of radioactivity from the bile was less than a tenth of the injected dose. The result of our study revealed that α-amanitin, β-amanitin, and phallacidin in the bile accounted for less than 20% of the total excretion in the urine and bile: <10% and <20% in the 20 and 60 mg/kg with biliary drainage groups. This, at first, seems a rather small amount. However, it needs to be considered that amatoxins in the bile are secreted by liver cells, which comes from the active toxin of hepatotoxicity. Additionally, hepatocytes were already damaged when amatoxins in the bile were reabsorbed and recirculated through the liver; the amatoxins in the bile may aggravate hepatocyte damage.

Activated charcoal should theoretically adsorb amatoxins that excreted via the bile into the duodenum [29]. Administration of activated charcoal not only adsorbs amatoxins eliminated via the bile, but also those secreted by the intestinal mucosa [8]. Serial charcoal dosing either as a continuous nasogastric drip or pulse dosing with 20–40 g every 3–4 h (for 24 h or more) has been advocated by most authors as a relatively noninvasive enterohepatic and enteric dialysis technique [30,31,32]. However, the relevance between this oral detoxication method and clinical outcome remained unclear [29].

## 4. Conclusions

This study is the first to establish a long-term bile drainage model with beagles in a normal state. This model can be applied to study bile secretion and components in bile-related diseases including amatoxin poisoning. After *Amanita exitialis* ingestion at the same dose, the beagles with biliary drainage showed less severe toxicity signs and biochemical and pathological changes and much lower internal exposure and accumulated amounts of peptide toxins in the urine than beagles without biliary drainage. Biliary drainage produced a more than 70% reduction in intestinal amatoxin absorption. This indicates that bile has a promoting effect on intestinal absorption of amatoxins. Amatoxins in the 0–2-day urine accounted for more than 90% of the total urine excretion, suggesting that enhanced amatoxin excretion in the urine may occur within two days after *Amanita exitialis* ingestion. Although amatoxins in bile accounted for less than 20% of the total excretion in the urine and bile, biliary drainage may be effective because amatoxins in the bile are the active toxins causing hepatotoxicity and may aggravate hepatocyte damage.

There are some limitations of our present study. First, the use of capsule form instead of toxins in a mushroom matrix could affect the absorption time and results. The disintegration of starch capsule in artificial gastric juice occurred within 15 min. Thus, we thought the effect caused by capsule was within an acceptable level. Second, we did not study the toxicokinetics of virotoxins. Third, we did not detect the contents of amatoxins in feces and organs for the lack of appropriate detection method; therefore, we could not study the distribution and excretion of amatoxins. Finally, bile drainage operation was performed before the beagles ingested *Amanita exitialis*; however, in practice, bile drainage operation is only conducted after the patient has ingested the poison. Therefore, we did not know the effects of bile drainage in patients poisoned with amatoxins.

## 5. Materials and Methods

### 5.1. Animals and Surgical Procedures

The study was approved by National Institute of Occupational Health and Poison Control review board for animal experiments. Twenty-four male beagles (age, 8–10 months; weight, 10 ± 1 kg) were obtained from Beijing Institute of Xieerxin Biology Resource (Beijing, China). They were randomly divided into four groups of six animals each: control, biliary drainage, and 20 and 60 mg/kg with biliary drainage groups. The biliary drainage operation was conducted in all groups barring the control group. The dogs were dressed in homemade underwear, coats, and Elizabeth circles for a week to ensure pre-experimental acclimatization (Figure S8a–c). After determining the baseline values for biochemical indicators, the dogs were fasted for 24 h but received water ad libitum. They were anesthetized by intravenous administration of pentobarbital sodium (3%, 1 mL/kg) during the surgery. A temperature of 38–39 °C was maintained with a warming mat. After skin preservation and sterilization, the abdominal cavity was opened through the central abdomen. The abdomen was explored and blunt dissection of the common bile duct was performed (Figure S8d). The common bile duct and its branches were ligated from the intestinal side. A 7.0-Fr drainage catheter (COOK, Bloomington, IN, USA) was percutaneously inserted into the gallbladder, and a medical drainage bag was connected to the drainage catheter coming out of the abdominal cavity. Each opened layer, including the peritoneum, abdominal wall muscle, and skin, was closed with suture. The operative incision was protected with sterile gauze and a bandage, and the dogs were dressed in homemade underwear, coats, and Elizabeth circles. The drainage bag was placed in the pocket of the coat (Figure S8e). After the anesthetic wore off, the dogs were placed in cages, and food and water were provided ad libitum. The operative incision was sterilized with iodophor twice a day until stitches were removed six days after surgery. The drainage bag was replaced every day. On the eighth day after surgery, the 20 and 60 mg/kg with biliary drainage groups were orally administered a lyophilized powder of *Amanita exitialis* in starch capsules. In control and bile drainage group, the beagle dogs were fed with empty capsules. Immediately after dosing, the animals were placed in their cages, and food and water were provided ad libitum. Dogs were sacrificed with an overdose of sodium pentobarbital at the end of the experiment. All procedures were performed according to the ethical standards of the National Institutes of Health Guide for Animal Welfare and approved by the Institutional Animal Care and Use Committee (EAWE-2017-005).

### 5.2. Chemicals

lyophilized *Amanita exitialis* (100 g) was provided by Professor Chen (College of Life Sciences, Hunan Normal University, Changsha, China). The *Amanita exitialis* powder was stored in a refrigerator at 2–8 °C. α-amanitin, β-amanitin, γ-amanitin, phallacidin, HPLC-grade water, acetonitrile, and ammonium acetate were obtained from Sigma-Aldrich (St. Louis, MO, USA). Normal saline (0.9% NaCl), ethyl alcohol, formalin, and hematoxylin and eosin were obtained from Sangon Biotech (Shanghai, China).

### 5.3. Peptide Toxins in Amanita exitialis

An ultra-performance liquid chromatography–electrospray ionization–tandem mass spectrometry (UPLC-ESI-MS/MS) method described previously was used to detect the peptide toxins in *Amanita exitialis* [16].

### 5.4. Toxicology Study

A toxicology study including an assessment of toxicity signs, a biochemical analysis of the blood, and a histopathological examination were performed as described previously [16].

### 5.5. Analysis of Peptide Toxins by UPLC-ESI-MS/MS

Peptide toxins in blood and urine samples were analyzed as described previously [16]. Bile samples from Days 0–1, Days 1–2, Days 2–3, and Days 3–4 were collected and stored in medical drainage bags and the bile volume was recorded. Plasma and urine samples were stored at 2–8 °C until analysis for peptide toxins.

#### 5.5.1. Extraction and Clean Up

α-Amanitin, β-amanitin, γ-amanitin, and phallacidin were used as the standard reference materials. Aliquots (0.2 mL) of bile samples were transferred into 2-mL polypropylene centrifuge tubes in duplicates. Next, 1 mL of acetonitrile was added to each tube. After vortex-mixing for 1 min, each sample was sonicated for 30 min and centrifuged at 11,200× *g* for 10 min at 4 °C. The supernatants were loaded on solid-phase extraction columns (Oasis PRiME HLB, 60 mg, 3 cc). The columns were then washed with 3 mL of methanol/ultrapure water (*v*/*v*: 5/95). The eluent was evaporated in a stream of nitrogen at room temperature. The dry residue was dissolved in 0.2 mL of ultrapure water and analyzed by UPLC-ESI-MS/MS.

#### 5.5.2. UPLC and MS/MS Parameters

UPLC and MS/MS parameters were as described in our previous study [16]. The limit of detection and limit of quantitation of each toxin in the bile were 0.03 mg/kg and 0.5 mg/kg, respectively.

### 5.6. Toxicokinetic Parameters and Statistical Analysis of Data

Toxicokinetic parameters were described in our previous study [16]. Measurement data are reported as mean ± standard deviation values and enumeration data are reported as percentage (%). Tukey’s honest significant difference test was used to identify the mean values that were significantly different between 20 and 60 mg/kg with biliary drainage groups. SPSS 22.0 (IBM Corporation, Armonk, NY, USA, 2015) was used for statistical analysis. The criterion for statistical significance was *p* ≤ 0.05. Except for the biliary drainage operation, the animals and methods in our present study are in accordance with our previously published study [16]. Thus, by directly comparing the data from this study with those from our published studies on *Amanita exitialis*-exposed beagles without biliary drainage, we can evaluate the impact of biliary drainage on the toxicity and toxicokinetics of *Amanita exitialis* in beagles.

## Figures and Tables

**Table 1:** Peptide toxin concentrations in *Amanita exitialis* (mg/kg dry weight).

Peptide Toxin	Toxin Concentration (mg/kg)					Mean	Standard Deviation (SD)
	1	2	3	4	5	(mg/kg)	(mg/kg)
α-amanitin	1934.6	1996.8	1891.4	2060	1947.8	1966.1	64.6
β-amanitin	992.6	995.2	857.4	902.9	827.2	915.1	76.9
γ-amanitin	8.6	8.5	8.6	8.2	8.7	8.5	0.2
phallacidin	618.6	636.1	575.8	672.7	508.2	602.3	63.1
Total	3554.4	3636.6	3333.2	3643.8	3291.9	3492	168.2

**Table 2:** Signs of toxicity and death in beagles following ingestion of *Amanita exitialis*.

Toxic Signs	Number of Dogs (%)		Time from Ingestion to Onset (h)	
	20 mg/kg with Biliary Drainage Group	60 mg/kg with Biliary Drainage Group	20 mg/kg with Billary Drainage Group	60 mg/kg with Billary Drainage Group
Loss of appetite	0 (0%)	3 (50%)	-	12–48
Vomiting	0 (0%)	3 (50%)	-	12–24
Diarrhea	0 (0%)	3 (50%)	-	12–24
Weakness	0 (0%)	0 (0%)	-	-
Hematemesis	0 (0%)	0 (0%)	-	-
Hematochezia	0 (0%)	0 (0%)	-	-
Death	0 (0%)	0 (0%)	-	-

**Table 3:** Toxicokinetic parameters for peptide toxins of beagles in different experimental groups

Parameter	α-Amanitin		β-Amanitin		Phallacidin	
	20 mg/kg with Biliary Drainage Group	60 mg/kg with Biliary Drainage Group	20 mg/kg with Biliary Drainage Group	60 mg/kg with Biliary Drainage Group	20 mg/kg with Biliary Drainage Group	60 mg/kg with Biliary Drainage Group
T1/2 (h)	1.08 ± 0.76	1.2 ± 0.58	1.29 ± 1.07	1.54 ± 0.71	-	0.62 ± 0.32 ^b,^*
AUC (0–∞) (µg/L × h)	5.36 ± 3.17	31.99 ± 9.65 ^a,^*	3.24 ± 1.49	15.75 ± 4.27 ^a,b^	-	2.08 ± 2.06 ^b,^*
Dose normalized AUC (0–∞)	138.9 ± 82.1	238.2 ± 83.9	179 ± 82.4	222.3 ± 79.2	-	60.5 ± 60.2 ^b,^*
C_max_ (µg/L)	1.73 ± 0.58	12.25 ± 3.54 ^a,^*	0.85 ± 0.33	5.18 ± 1.32 ^a,b^	-	0.85 ± 0.37 ^b,^*
Dose normalized C_max_	44 ± 15.1	86.1 ± 30.8	57 ± 18.3	96 ± 24.4	-	24.8 ± 10.8 ^b,^*
T_max_ (h)	1.38 ± 0.48	1.13 ± 0.25	1.63 ± 0.48	1.25 ± 0.29	-	1.38 ± 0.25
CL/F (L/h/kg)	0.85 ± 0.43	0.36 ± 0.11	0.64 ± 0.42	0.33 ± 0.09	-	1.1 ± 0.86 ^b,^*
V_z_/F (L/kg)	1.07 ± 0.31	0.68 ± 0.48	0.78 ± 0.63	1.38 ± 0.59	-	1.77 ± 0.57 ^b,^*
MRT (0–∞) (h)	3.55 ± 0.8	2.87 ± 0.43	3.83 ± 1.02	3.89 ± 0.46	-	1.95 ± 0.73 ^b,^*

We could not calculate the toxicokinetic parameters because phallacidin was not detected in serum in the 20 mg/kg with biliary drainage group. ^a^ indicates *p* ≤ 0.05 when compared to the same toxin in the 20 mg/kg with biliary drainage group. ^b^ indicates *p* ≤ 0.05 when compared to α-amanitin in the same experimental group. * indicates *p* ≤ 0.05 when compared to β-amanitin in the same experimental group.

**Table 4:** The daily amounts of peptide toxins excreted in urine in beagles with biliary drainage (mg).

Time after Ingestion	α-Amanitin		β-Amanitin		Phallacidin	
	20 mg/kg with Biliary Drainage Group	60 mg/kg with Biliary Drainage Group	20 mg/kg with Biliary Drainage Group	60 mg/kg with Biliary Drainage Group	20 mg/kg with Biliary Drainage Group	60 mg/kg with Biliary Drainage Group
0–1 days	0.0147 (96.3%)	0.0213 (90.9%)	0.0050 (93.9%)	0.0065 (91.4%)	0.0012 (92.2%)	0.0017 (70.5%)
1–2 days	0.0004 (3.7%)	0.0007 (8.9%)	0.0005 (6.1%)	0.0003 (8.6%)	0.0001 (7.8%)	0.0005 (22.1%)
2–3 days	0(0%)	0.0001 (0.2%)	0(0%)	0(0%)	0(0%)	0.0002 (7.5%)
3–4 days	0(0%)	0(0%)	0(0%)	0(0%)	0(0%)	0(0%)
Total	0.0151 (100%)	0.0222 (100%)	0.0055 (100%)	0.0068 (100%)	0.0014 (100%)	0.0025 (100%)

The data in parentheses are the percentages of peptide toxins excreted daily in bile.

**Table 5:** The daily amounts of peptide toxins excreted in bile in beagles with biliary drainage (mg).

Time after Ingestion	α-Amanitin		β-Amanitin		Phallacidin	
	20 mg/kg with Biliary Drainage Group	60 mg/kg with Biliary Drainage Group	20 mg/kg with Biliary Drainage Group	60 mg/kg with Biliary Drainage Group	20 mg/kg with Biliary Drainage Group	60 mg/kg with Biliary Drainage Group
0–1 days	0.00007 (100%)	0.00061 (100%)	0.00014 (100%)	0.00122 (100%)	0.00005 (100%)	0.00020 (81.7%)
1–2 days	0(0%)	0(0%)	0(0%)	0(0%)	0(0%)	0.00004 (18.3%)
2–3 days	0(0%)	0(0%)	0(0%)	0(0%)	0(0%)	0(0%)
3–4 days	0(0%)	0(0%)	0(0%)	0(0%)	0(0%)	0(0%)
Accumulated amounts	0.00007 (100%)	0.00061 (100%)	0.00014 (100%)	0.00122 (100%)	0.00005 (100%)	0.00024 (100%)

The data in parentheses are the percentages of peptide toxins excreted daily in bile.

**Table 6:** The ratio of peptide toxins in bile to excretion in urine and bile.

Peptide Toxin	20 mg/kg with Biliary Drainage Group (%)	60 mg/kg with Biliary Drainage Group (%)
α-Amanitin	1.3 ± 2.4	3.1 ± 2.5
β-Amanitin	5.7 ± 8.5	15.4 ± 10.6
Phallacidin	4.8 ± 5.7	9.4 ± 6.4

## Supplementary Materials

Supplementary Materials are available online at http://www.mdpi.com/2072-6651/10/6/215/s1. Figure S1: Images showing the livers of beagle dogs that died 72 h in different experimental groups, Figure S2: Representative photomicrographs of H&E-stained sections of the liver of beagle dogs that died 72 h in different experimental groups, Figure S3: Representative photomicrographs of H&E-stained sections of the liver of beagle dogs that died 72 h in different experimental groups, Figure S4: The urine and bile volumes in different experimental groups, Figure S5: Changes in ALT, AST, TBIL, and DBIL level in beagles in different experimental groups, Figure S6: Changes in ALP, PT, APTT and INR values in beagles in different experimental groups, Figure S7: Changes in plasma concentrations of amatoxins in beagles in different experimental groups, Figure S8: (a) Underwear (b) Coat and (c) Elizabeth circle wore in beagles. (d) Common bile duct of beagles (e) Pocket of the coat wore in beagles, Table S1: The amount of peptide toxins in *A. exitialis* ingested in different experimental groups, Table S2: Dynamic changes in coagulation, hepatic and renal function indicator of beagles in biliary drainage group, Table S3: The detection rate of peptide toxins in blood of beagles (%), Table S4: The toxin contents in urine of beagles in different experimental groups (μg/L), Table S5: The detection rate of peptide toxins in urine of beagles (%), Table S6: The toxin concentrations in bile of beagles ingested of *Amanita exitialis* (μg/L), Table S7: The detection rate of peptide toxins in bile of beagles (%), Table S8: The daily amounts of peptide toxins excreted in urine in beagles without biliary drainage(mg).
