# Supplementary material for: Effect of Biliary Drainage on the Toxicity and Toxicokinetics of Amanita exitialis in Beagles
Source: Toxins (Basel). 2018 May 25;10(6):215. doi: 10.3390/toxins10060215 (PMC6024615; doi:10.3390/toxins10060215)
Supplement: Supplementary file 1 [file toxins-10-00215-s001.pdf]

Supplementary Materials: Effect of biliary drainage on the toxicity and toxicokinetics of *Amanita exitialis* in beagles

Jian Sun, Yu-Min Niu, Yu-Tao Zhang, Hai-Jiao Li, Yu Yin, Yi-Zhe Zhang, Pei-Bin Ma, Jing Zhou, Jun-Jia Lu, Hong-Shun Zhang and Cheng-Ye Sun

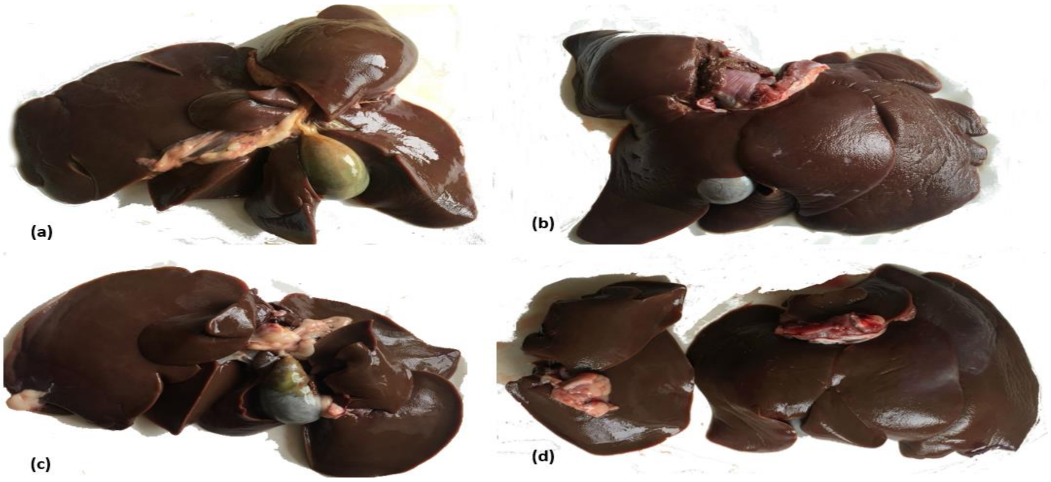

Figure S1. Images showing the livers of beagle dogs that died 72 h in different experimental groups. (**a**) In control group. (**b**) In biliary drainage group. (**c**) 20 mg/kg with biliary drainage group. (**d**) 60 mg/kg with biliary drainage group.

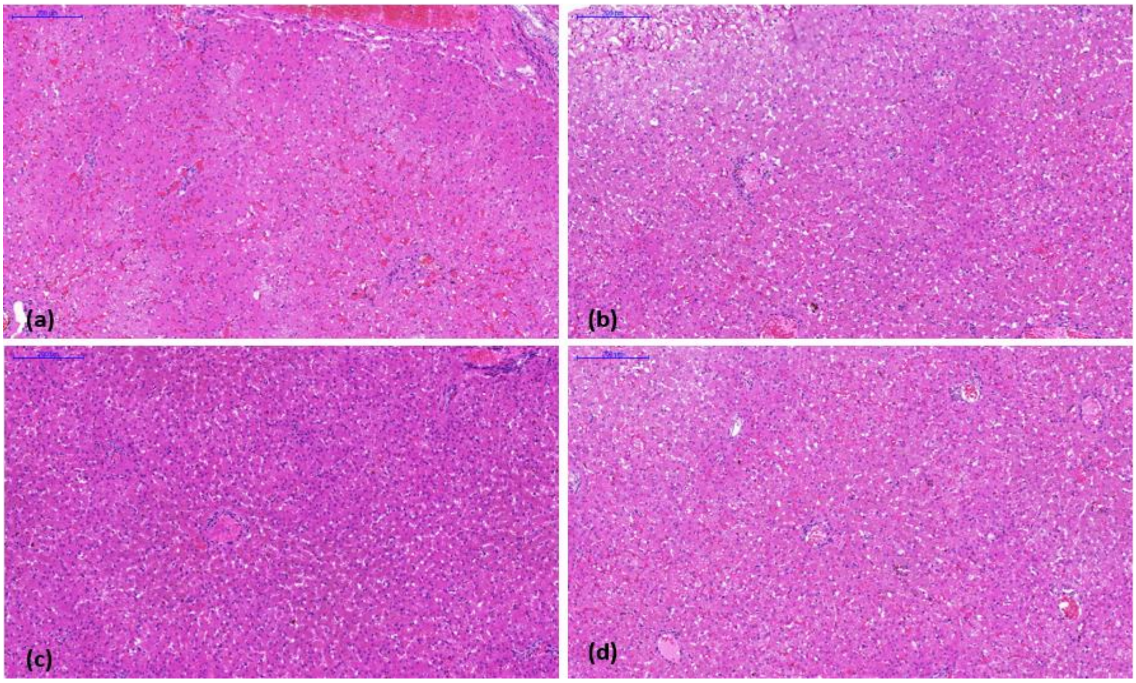

Figure S2. Representative photomicrographs of H&E-stained sections of the liver of beagle dogs that died 72 h in different experimental groups. (**a**) In control group. (**b**) In biliary drainage group. (**c**) 20 mg/kg with biliary drainage group. (**d**) 60 mg/kg with biliary drainage group. Original magnification, 200 ×.

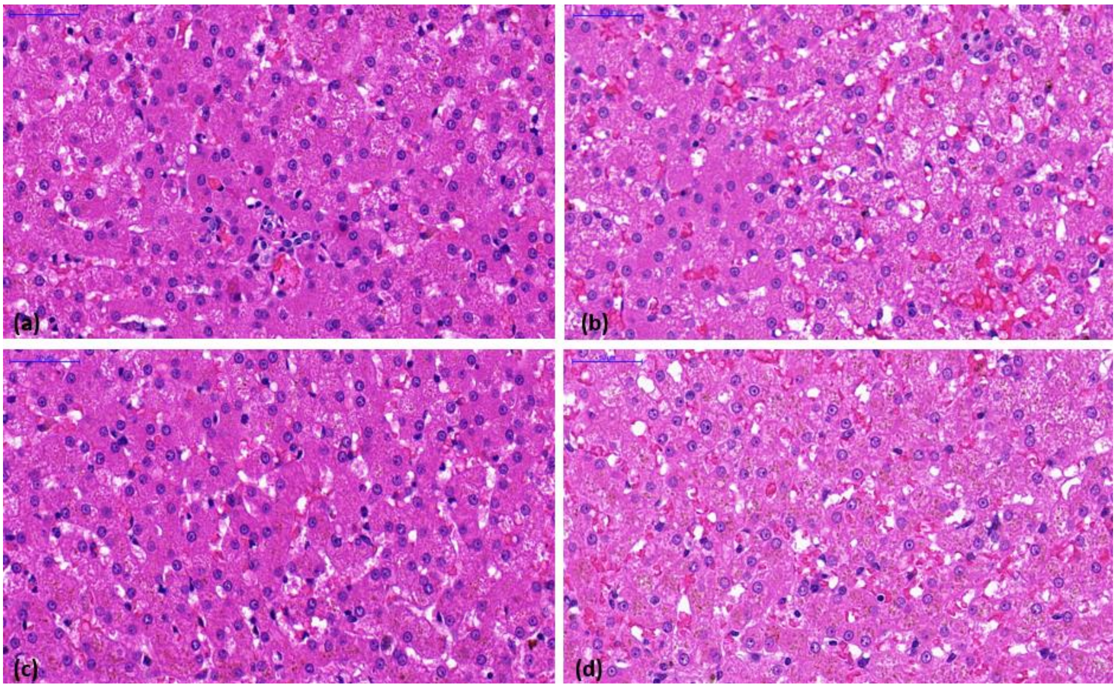

Figure S3. Representative photomicrographs of H&E-stained sections of the liver of beagle dogs that died 72 h in different experimental groups. (**a**) In control group. (**b**) In biliary drainage group. (**c**) 20 mg/kg with biliary drainage group. (**d**) 60 mg/kg with biliary drainage group. Original magnification, 400 ×.

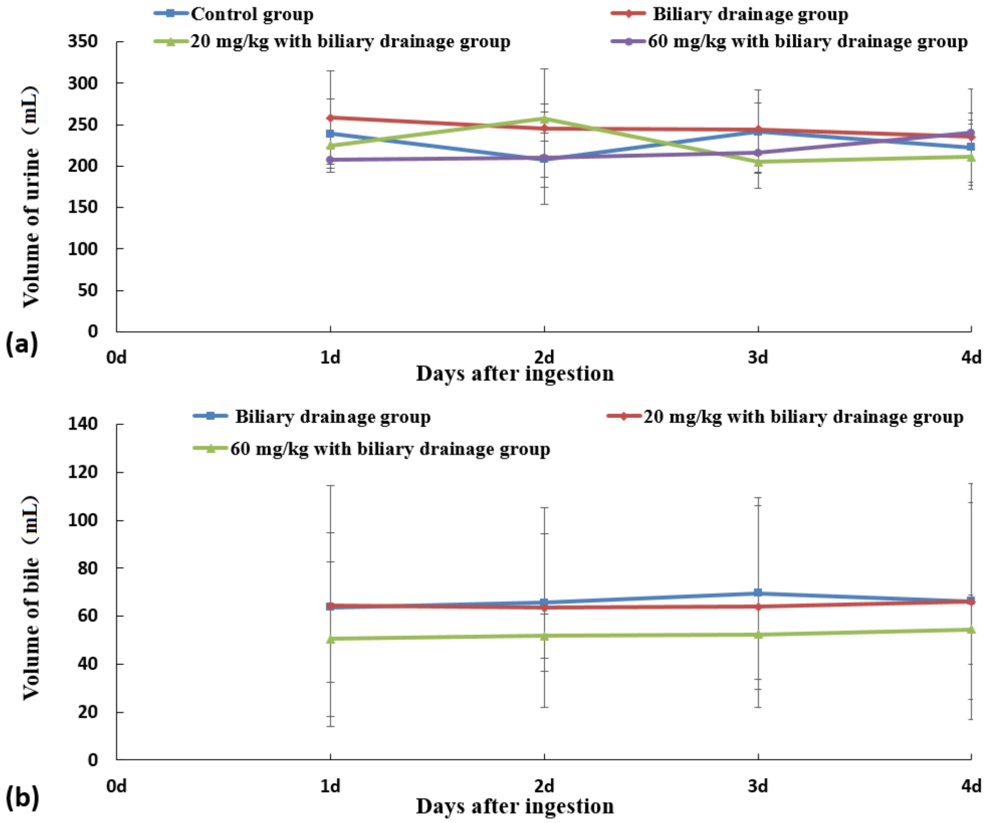

Figure S4. The urine and bile volumes in different experimental groups. (**a**) The urine volume in different experimental groups and (**b**) The bile volume in different experimental groups.

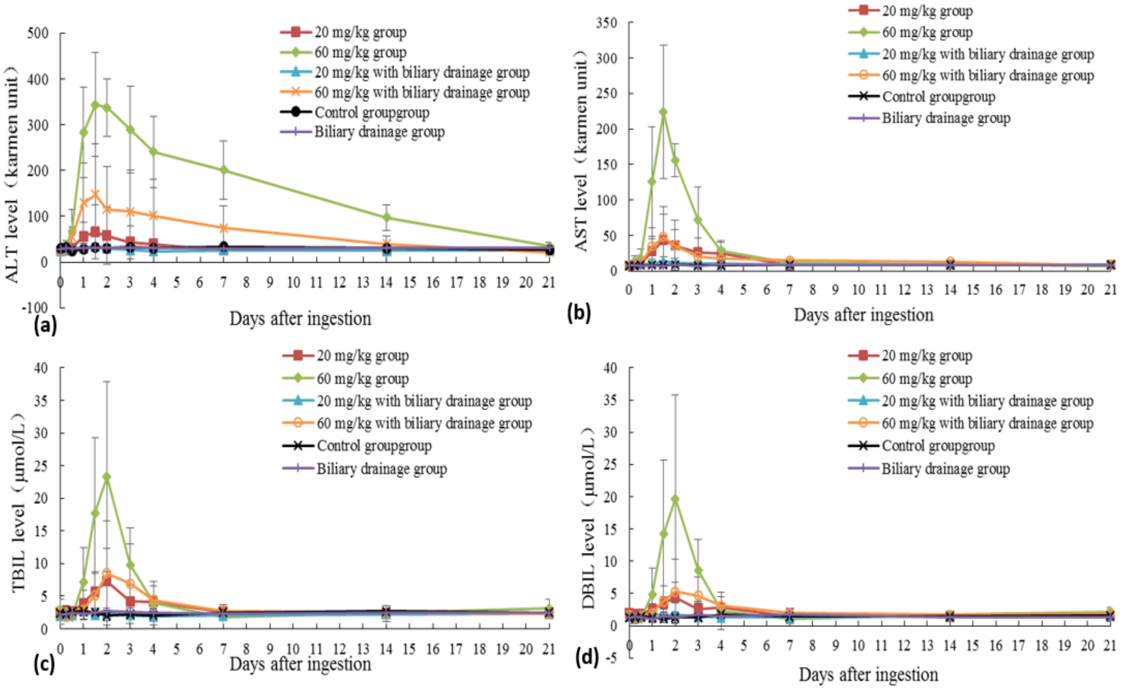

Figure S5. Changes in ALT, AST, TBIL, and DBIL level in beagles in different experimental groups.

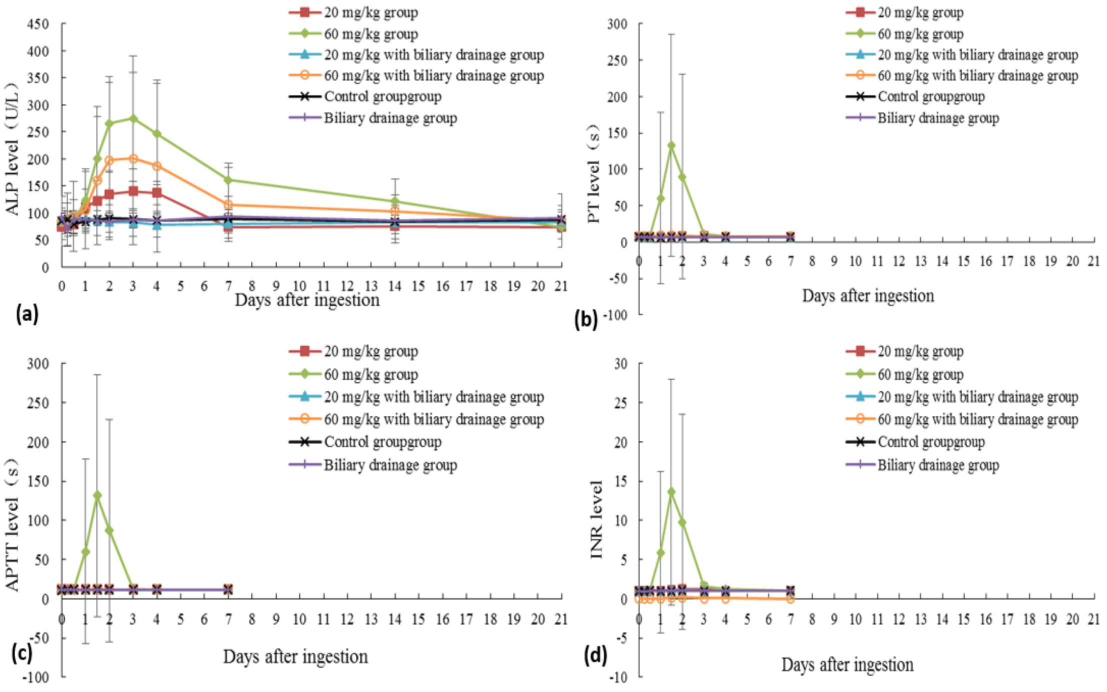

Figure S6. Changes in ALP, PT, APTT and INR values in beagles in different experimental groups.

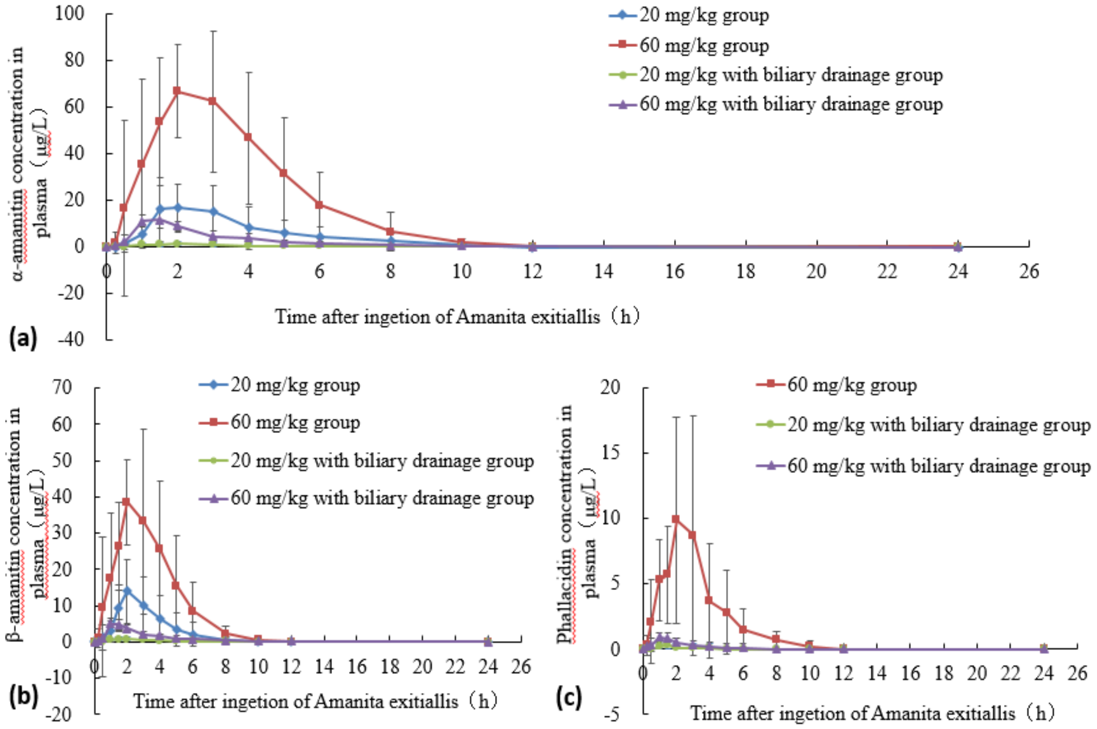

Figure S7. Changes in plasma concentrations of amatoxins in beagles in different experimental groups.

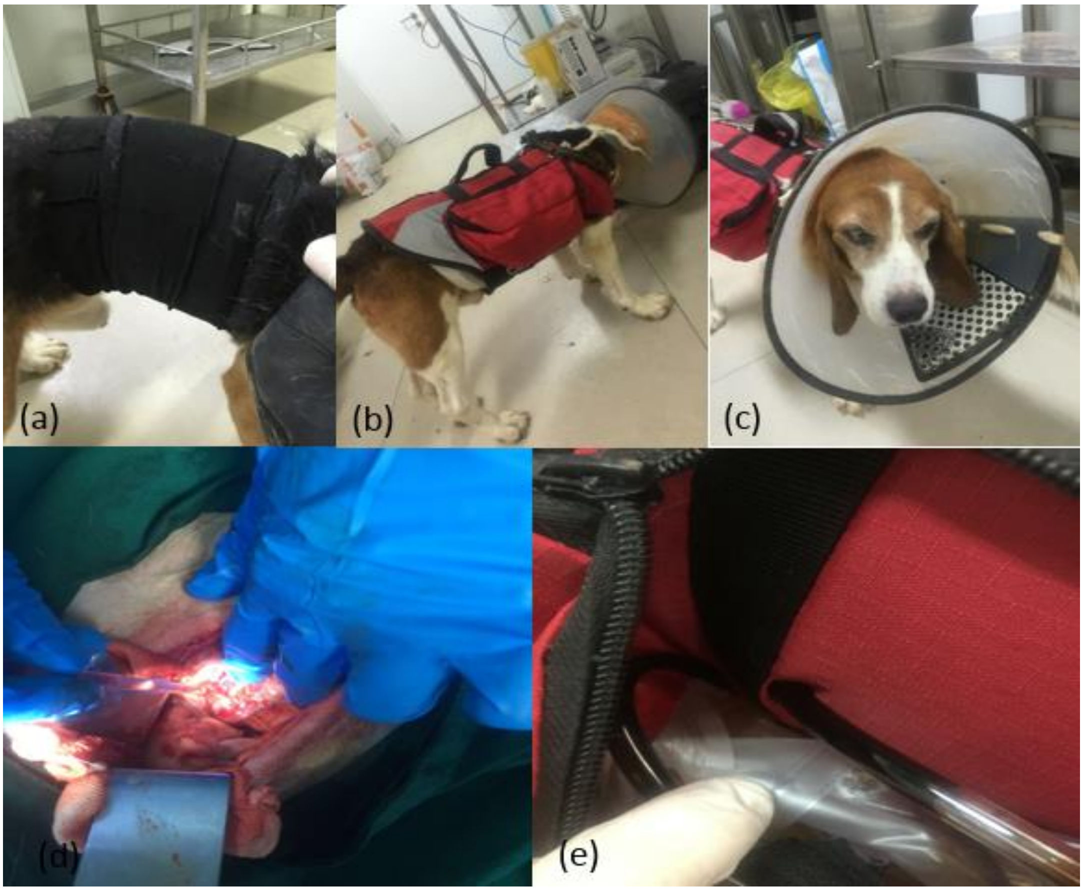

Figure S8. (**a**) Underwear (**b**) Coat and (**c**) Elizabeth circle wore in beagles. (**d**) Common bile duct of beagles (**e**) Pocket of the coat wore in beagles.

Table S1. The amount of peptide toxins in *A. exitialis* ingested in different experimental groups.

Peptide toxin	20 mg/kg (mg)	60 mg/kg (mg)	20 mg/kg with biliary drainage group (mg)	60 mg/kg with biliary drainage group (mg)
α-Amanitin	0.3891 ± 0.0018	1.1673 ± 0.0055	0.3855 ± 0.0021	1.1473 ± 0.0075
β-Amanitin	0.1843 ± 0.0012	0.5530 ± 0.0035	0.1809 ± 0.0032	0.5392 ± 0.0072
Phallacidin	0.1214 ± 0.0008	0.3643 ± 0.0024	0.1198 ± 0.0010	0.3428 ± 0.0047

Table S2. Dynamic changes in coagulation, hepatic and renal function indicator of beagles in biliary drainage group.

Time after surgery	ALT (Kar-men unit)	AST (Kar-men unit)	TBIL (μmol/L)	DBIL (μmol/L)	ALP (U/L)	PT(s)	APTT (s)	INR	BUN (mmol/L)	CRE (mmol/L)
0d	30.7 ± 6.1	7.5 ± 1	2.3 ± 0.8	1.1 ± 0.4	83.1 ± 16.8	7.2 ± 0.2	11.3 ± 0.1	1 ± 0	5.9 ± 1.1	85.7 ± 10.8
1d	42.6 ± 2.6	8.5 ± 2.2	2.4 ± 0.7	1.8 ± 0.4	92.9 ± 15.1	7.1 ± 0.4	11.3 ± 0.1	1 ± 0	6.5 ± 1.4	88.4 ± 13.5
2d	60.4 ± 8.9	11.9 ± 4.5	2.3 ± 0.7	1.2 ± 0.6	105.7 ± 23.9	7.1 ± 0.3	11.3 ± 0	1 ± 0	6.2 ± 1.5	90.5 ± 16.6
3d	47.8 ± 5.9	10 ± 3.1	2.3 ± 0.9	1.5 ± 0.6	103.8 ± 31.8	7 ± 0.4	11.2 ± 0.1	1 ± 0.1	6.1 ± 1.1	85 ± 16.1
4d	36.7 ± 4.8	8.5 ± 1.7	2.1 ± 0.8	1.5 ± 0.4	97 ± 22.2	7.1 ± 0.2	11.3 ± 0.1	1 ± 0.1	6.9 ± 0.8	86.5 ± 8.5
7d	28.6 ± 6.8	7.8 ± 1	2.2 ± 0.6	1.2 ± 0.2	92.3 ± 12.2	7.1 ± 0.2	11.3 ± 0.1	1 ± 0	6.1 ± 1.7	85.7 ± 10.2

Table S3. The detection rate of peptide toxins in blood of beagles (%).

Time after ingestion (h)	α-Amanitin		β-Amanitin		Phallacidin	
	20 mg/kg with biliary drainage group	60 mg/kg with biliary drainage group	20 mg/kg with biliary drainage group	60 mg/kg with biliary drainage group	20 mg/kg with biliary drainage group	60 mg/kg with biliary drainage group
0	0	0	0	0	0	0
0.25	0	0	0	50	0	33
0.5	33	33	33	66	0	33
1	100	100	100	100	50	100
1.5	100	100	100	100	66	100
2	100	100	100	100	33	100
3	100	100	100	100	33	50
4	100	100	100	100	17	50
5	100	100	100	100	0	33
6	100	100	83	100	0	17
8	50	50	83	100	0	0
10	50	50	66	100	0	0
12	17	33	33	100	0	0
24	0	0	0	33	0	0
36	0	0	0	0	0	0
48	0	0	0	0	0	0

Table S4. The toxin contents in urine of beagles in different experimental groups (μg/L).

**Time after ingestion**	**α-Amanitin**		**β-Amanitin**		**Phallacidin**	
	20 mg/kg with biliary drainage group	60 mg/kg with biliary drainage group	20 mg/kg with biliary drainage group	60 mg/kg with biliary drainage group	20 mg/kg with biliary drainage group	60 mg/kg with biliary drainage group
0-1d	69.9 ± 42.4	128.4 ± 98.1	25.3 ± 25.7	36.7 ± 14.1	6.2 ± 4.1	9.7 ± 8.3
1-2d	1.6 ± 1.2	1.3 ± 1.1	1.9 ± 2.8	0.6 ± 1.5	0.5 ± 0.8	3.4 ± 1.6
2-3d	0	0.5 ± 0.4	0	0	0	1.5 ± 1.3
3-4d	0	0	0	0	0	0

Table S5. The detection rate of peptide toxins in urine of beagles (%).

**Time after ingestion**	**α-Amanitin**		**β-Amanitin**		**Phallacidin**	
	20 mg/kg with biliary drainage group	60 mg/kg with biliary drainage group	20 mg/kg with biliary drainage group	60 mg/kg with biliary drainage group	20 mg/kg with biliary drainage group	60 mg/kg with biliary drainage group
0-1 d	100	100	100	100	100	100
1-2 d	100	100	50	83	83	100
2-3 d	0	50	0	0	0	50
3-4 d	0	0	0	0	0	0

Table S6. The toxin concentrations in bile of beagles ingested of *Amanita exitialis* (μg/L).

**Time after ingestion**	**α-Amanitin**		**β-Amanitin**		**Phallacidin**	
	20 mg/kg with biliary drainage group	60 mg/kg with biliary drainage group	20 mg/kg with biliary drainage group	60 mg/kg with biliary drainage group	20 mg/kg with biliary drainage group	60 mg/kg with biliary drainage group
0-1d	1.2 ± 1.4	12.4 ± 12.1	2.2 ± 2.8	26.7 ± 26.4	0	0.8 ± 0.7
1-2d	0	0	0	0	0	0.8
2-3d	0	0	0	0	0	0
3-4d	0	0	0	0	0	0

Table S7. The detection rate of peptide toxins in bile of beagles (%).

**Time after ingestion**	**α-Amanitin**		**β-Amanitin**		**Phallacidin**	
	20 mg/kg with biliary drainage group	60 mg/kg with biliary drainage group	20 mg/kg with biliary drainage group	60 mg/kg with biliary drainage group	20 mg/kg with biliary drainage group	60 mg/kg with biliary drainage group
0-1 d	50	83	50	83	83	83
1-2 d	0	0	0	0	0	75
2-3 d	0	0	0	0	0	0
3-4 d	0	0	0	0	0	0

Table S8. The daily amounts of peptide toxins excreted in urine in beagles without biliary drainage(mg).

**Time after ingestion**	**α-Amanitin**		**β-Amanitin**		**Phallacidin**	
	20 mg/kg group	60 mg/kg group	20 mg/kg group	60 mg/kg group	20 mg/kg group	60 mg/kg group
0-1d	0.0379 (97.9%)	0.0593 (92.8%)	0.0156 (99.9%)	0.0251 (94.7%)	0.0035 (94.2%)	0.0088 (76%)
1-2d	0.0388 (99.995%)	0.0618 (97.5%)	0.01567 (100%)	0.0255 (96.2%)	0.0037 (100%)	0.0110 (93%)
2-3d	0.0002 (100%)	0.0626 (99.4%)	0 (100%)	0.0260 (98.2%)	0 (100%)	0.0115 (97.3%)
3-4d	0 (100%)	0.0630 (100%)	0 (100%)	0.0264 (100%)	0(100%)	0.0118 (100%)
Accumul-ative amount	0.0390 (100%)	0.0630 (100%)	0.01567 (100%)	0.0264 (100%)	0.0037 (100%)	0.0118 (100%)
